# Supplementary material for: Pyruvate Dehydrogenase Kinase 1 inhibition mediated oxidative phosphorylation enhancement in cartilage promotes osteoarthritis progression
Source: BMC Musculoskelet Disord. 2023 Jul 20;24:597. doi: 10.1186/s12891-023-06585-6 (PMC10357736; doi:10.1186/s12891-023-06585-6)
Supplement: Supplementary file 1 — Additional file 1. [file 12891_2023_6585_MOESM1_ESM.zip › BP.Gsea.1653623667859/GOBP_REGULATION_OF_RELEASE_OF_CYTOCHROME_C_FROM_MITOCHONDRIA.html]

Details for gene set GOBP\_REGULATION\_OF\_RELEASE\_OF\_CYTOCHROME\_C\_FROM\_MITOCHONDRIA[GSEA]

|  || Dataset | 55457\_collapsed\_to\_symbols.cls.cls #OA\_versus\_normal.cls.cls #OA\_versus\_normal\_repos |
| Phenotype | cls.cls#OA\_versus\_normal\_repos |
| Upregulated in class | OA |
| GeneSet | GOBP\_REGULATION\_OF\_RELEASE\_OF\_CYTOCHROME\_C\_FROM\_MITOCHONDRIA |
| Enrichment Score (ES) | 0.41880932 |
| Normalized Enrichment Score (NES) | 1.64526 |
| Nominal p-value | 0.0071599046 |
| FDR q-value | 0.21643142 |
| FWER p-Value | 1.0 |
Table: GSEA Results Summary

  

Fig 1: Enrichment plot: GOBP\_REGULATION\_OF\_RELEASE\_OF\_CYTOCHROME\_C\_FROM\_MITOCHONDRIA      
 Profile of the Running ES Score & Positions of GeneSet Members on the Rank Ordered List

  

| SYMBOL | TITLE | RANK IN GENE LIST | RANK METRIC SCORE | RUNNING ES | CORE ENRICHMENT || 1 | FAM162A | family with sequence similarity 162 member A [Source:HGNC Symbol;Acc:HGNC:17865] | 4 | 1.258 | 0.0851 | Yes |
| 2 | PARL | presenilin associated rhomboid like [Source:HGNC Symbol;Acc:HGNC:18253] | 14 | 1.156 | 0.1629 | Yes |
| 3 | PSMD10 | "proteasome 26S subunit, non-ATPase 10 [Source:HGNC Symbol;Acc:HGNC:9555]" | 58 | 0.976 | 0.2258 | Yes |
| 4 | GHITM | growth hormone inducible transmembrane protein [Source:HGNC Symbol;Acc:HGNC:17281] | 213 | 0.809 | 0.2688 | Yes |
| 5 | MFF | mitochondrial fission factor [Source:HGNC Symbol;Acc:HGNC:24858] | 932 | 0.531 | 0.2490 | Yes |
| 6 | PYCARD | PYD and CARD domain containing [Source:HGNC Symbol;Acc:HGNC:16608] | 1164 | 0.484 | 0.2639 | Yes |
| 7 | AKT1 | AKT serine/threonine kinase 1 [Source:HGNC Symbol;Acc:HGNC:391] | 1192 | 0.478 | 0.2942 | Yes |
| 8 | MOAP1 | modulator of apoptosis 1 [Source:HGNC Symbol;Acc:HGNC:16658] | 1215 | 0.473 | 0.3246 | Yes |
| 9 | TNFSF10 | TNF superfamily member 10 [Source:HGNC Symbol;Acc:HGNC:11925] | 1241 | 0.468 | 0.3545 | Yes |
| 10 | GPER1 | G protein-coupled estrogen receptor 1 [Source:HGNC Symbol;Acc:HGNC:4485] | 1677 | 0.402 | 0.3479 | Yes |
| 11 | PRKN | parkin RBR E3 ubiquitin protein ligase [Source:HGNC Symbol;Acc:HGNC:8607] | 1972 | 0.364 | 0.3498 | Yes |
| 12 | CLU | clusterin [Source:HGNC Symbol;Acc:HGNC:2095] | 2083 | 0.352 | 0.3651 | Yes |
| 13 | PINK1 | PTEN induced kinase 1 [Source:HGNC Symbol;Acc:HGNC:14581] | 2097 | 0.350 | 0.3878 | Yes |
| 14 | TRIAP1 | TP53 regulated inhibitor of apoptosis 1 [Source:HGNC Symbol;Acc:HGNC:26937] | 2163 | 0.342 | 0.4060 | Yes |
| 15 | IGF1 | insulin like growth factor 1 [Source:HGNC Symbol;Acc:HGNC:5464] | 2286 | 0.328 | 0.4188 | Yes |
| 16 | HGF | hepatocyte growth factor [Source:HGNC Symbol;Acc:HGNC:4893] | 2547 | 0.297 | 0.4188 | No |
| 17 | BIK | BCL2 interacting killer [Source:HGNC Symbol;Acc:HGNC:1051] | 3057 | 0.244 | 0.3958 | No |
| 18 | BAD | BCL2 associated agonist of cell death [Source:HGNC Symbol;Acc:HGNC:936] | 3788 | 0.178 | 0.3511 | No |
| 19 | CIDEB | cell death inducing DFFA like effector b [Source:HGNC Symbol;Acc:HGNC:1977] | 3992 | 0.157 | 0.3459 | No |
| 20 | PLSCR3 | phospholipid scramblase 3 [Source:HGNC Symbol;Acc:HGNC:16495] | 4557 | 0.109 | 0.3095 | No |
| 21 | BNIP3 | BCL2 interacting protein 3 [Source:HGNC Symbol;Acc:HGNC:1084] | 5501 | 0.032 | 0.2383 | No |
| 22 | TP53 | tumor protein p53 [Source:HGNC Symbol;Acc:HGNC:11998] | 5511 | 0.032 | 0.2397 | No |
| 23 | BCL2L2 | BCL2 like 2 [Source:HGNC Symbol;Acc:HGNC:995] | 5643 | 0.020 | 0.2309 | No |
| 24 | BID | BH3 interacting domain death agonist [Source:HGNC Symbol;Acc:HGNC:1050] | 5715 | 0.014 | 0.2264 | No |
| 25 | PLAUR | "plasminogen activator, urokinase receptor [Source:HGNC Symbol;Acc:HGNC:9053]" | 6362 | -0.032 | 0.1783 | No |
| 26 | BCL2L1 | BCL2 like 1 [Source:HGNC Symbol;Acc:HGNC:992] | 6698 | -0.055 | 0.1560 | No |
| 27 | MLLT11 | MLLT11 transcription factor 7 cofactor [Source:HGNC Symbol;Acc:HGNC:16997] | 7130 | -0.091 | 0.1287 | No |
| 28 | AVP | arginine vasopressin [Source:HGNC Symbol;Acc:HGNC:894] | 7201 | -0.096 | 0.1298 | No |
| 29 | ARRB2 | arrestin beta 2 [Source:HGNC Symbol;Acc:HGNC:712] | 7255 | -0.100 | 0.1324 | No |
| 30 | NOL3 | nucleolar protein 3 [Source:HGNC Symbol;Acc:HGNC:7869] | 7545 | -0.122 | 0.1182 | No |
| 31 | FXN | frataxin [Source:HGNC Symbol;Acc:HGNC:3951] | 8057 | -0.160 | 0.0893 | No |
| 32 | DNM1L | dynamin 1 like [Source:HGNC Symbol;Acc:HGNC:2973] | 8499 | -0.194 | 0.0682 | No |
| 33 | OPA1 | OPA1 mitochondrial dynamin like GTPase [Source:HGNC Symbol;Acc:HGNC:8140] | 8655 | -0.205 | 0.0700 | No |
| 34 | MMP9 | matrix metallopeptidase 9 [Source:HGNC Symbol;Acc:HGNC:7176] | 8698 | -0.209 | 0.0809 | No |
| 35 | PDCD5 | programmed cell death 5 [Source:HGNC Symbol;Acc:HGNC:8764] | 9039 | -0.235 | 0.0704 | No |
| 36 | BAX | "BCL2 associated X, apoptosis regulator [Source:HGNC Symbol;Acc:HGNC:959]" | 9323 | -0.257 | 0.0659 | No |
| 37 | PPIF | peptidylprolyl isomerase F [Source:HGNC Symbol;Acc:HGNC:9259] | 9999 | -0.321 | 0.0352 | No |
| 38 | PMAIP1 | phorbol-12-myristate-13-acetate-induced protein 1 [Source:HGNC Symbol;Acc:HGNC:9108] | 10084 | -0.329 | 0.0510 | No |
| 39 | HRK | "harakiri, BCL2 interacting protein [Source:HGNC Symbol;Acc:HGNC:5185]" | 10383 | -0.359 | 0.0522 | No |
| 40 | LMNA | lamin A/C [Source:HGNC Symbol;Acc:HGNC:6636] | 10946 | -0.424 | 0.0373 | No |
| 41 | PLA2G6 | phospholipase A2 group VI [Source:HGNC Symbol;Acc:HGNC:9039] | 11128 | -0.446 | 0.0535 | No |
| 42 | BCL2L11 | BCL2 like 11 [Source:HGNC Symbol;Acc:HGNC:994] | 11867 | -0.564 | 0.0344 | No |
| 43 | BBC3 | BCL2 binding component 3 [Source:HGNC Symbol;Acc:HGNC:17868] | 12298 | -0.676 | 0.0468 | No |
Table: GSEA details [plain text format]

  

Fig 2: GOBP\_REGULATION\_OF\_RELEASE\_OF\_CYTOCHROME\_C\_FROM\_MITOCHONDRIA      
 Blue-Pink O' Gram in the Space of the Analyzed GeneSet

  

Fig 3: GOBP\_REGULATION\_OF\_RELEASE\_OF\_CYTOCHROME\_C\_FROM\_MITOCHONDRIA: Random ES distribution      
 Gene set null distribution of ES for **GOBP\_REGULATION\_OF\_RELEASE\_OF\_CYTOCHROME\_C\_FROM\_MITOCHONDRIA**
